# Predicting Particulate Matter (*PM*_10_) Levels in Morocco: A 5-Day Forecast Using the Analog Ensemble Method

**DOI:** 10.21203/rs.3.rs-4619478/v1

**Published:** 2024-08-02

**Authors:** Anass Houdou, Kenza Khomsi, Luca Delle Monache, Weiming Hu, Saber Boutayeb, Lahcen Belyamani, Fayez Abdulla, Wael K. Al-Delaimy, Mohamed Khalis

**Affiliations:** 1 International School of Public Health, Mohammed VI University of Sciences and Health, Casablanca, Morocco; 2 Mohammed VI Center for Research & Innovation, Rabat, Morocco; 3 General Directorate of Meteorology, Mohammed VI University of Sciences and Health, Casablanca, Morocco; 4 Center for Western Weather and Water Extremes, Scripps Institution of Oceanography, University of California San Diego, San Diego, USA; 5 School of Integrated Sciences, James Madison University, Virginie, USA; 6 Faculty of Medicine, Mohammed VI University of Sciences and Health, Casablanca, Morocco; 7 Civil Engineering Department, Jordan University of Science and Technology, Irbid 22120, Jordan; 8 School of Public Health, University of California San Diego, La Jolla, CA 92093-0628, San Diego, USA; 9 Laboratory of Biostatistics, Clinical, and Epidemiological Research, & Laboratory of Community Health (Public Health, Preventive Medicine and Hygiene), Department of Public Health, Faculty of Medicine and Pharmacy, Mohammed V University in Rabat, Rabat, Morocco; 10 Higher Institute of Nursing Professions and Technical Health, Rabat, Morocco

**Keywords:** Air pollution, Particulate matter (*PM*_10_), Forecasting, Analog ensemble model, Copernicus atmosphere monitoring service, Morocco

## Abstract

Accurate prediction of Particulate Matter (*PM*_10_) levels, an indicator of natural pollutants such as those resulting from dust storms, is crucial for public health and environmental planning. This study aims to provide accurate forecasts of *PM*_10_ over Morocco for five days. The Analog Ensemble (AnEn) and the Bias Correction (AnEnBc) techniques were employed to post-process *PM*_10_ forecasts produced by the Copernicus Atmosphere Monitoring Service (CAMS) global atmospheric composition forecasts, using CAMS reanalysis data as a reference. The results show substantial prediction improvements: the Root Mean Square Error (RMSE) decreased from 63.83 *μg*/*m*^3^ in the original forecasts to 44.73 *μg*/*m*^3^ with AnEn and AnEnBc, while the Mean Absolute Error (MAE) reduced from 36.70 *μg*/*m*^3^ to 24.30 *μg*/*m*^3^. Additionally, the coefficient of determination (*R*^2^) increased more than twofold from 29.11% to 65.18%, and the Pearson correlation coefficient increased from 0.61 to 0.82. This is the first use of this approach for Morocco and the Middle East and North Africa and has the potential for translation into early and more accurate warnings of *PM*_10_ pollution events. The application of such approaches in environmental policies and public health decision making can minimize air pollution health impacts.

## Introduction

The world health organization has identified air pollution as one of the main environmental risks to human health. According to the World Health Statistics (2023), almost 99% of the worldwide population breathes unhealthy levels of fine particulate matter. Ambient and household air pollution have been associated with 6.7 million deaths annually worldwide in 2019. Ambient air pollution alone is responsible for 4.2 million deaths every year, specifically due to exposure to fine particulate matter, which leads to cardiovascular, cancer, and respiratory diseases (Chen et al., 2019; Yan et al., 2019). Morocco is no exception, with 7,034 deaths every year attributed to particulate matter (World Bank, 2019; World Bank and Institute for Health Metrics and Evaluation, 2016). Given that particulate matter originates from various sources (Abulude et al., 2019; Friend et al., 2013), predicting its presence is crucial for effective prevention.

Mo et al. (2019) have highlighted that providing accurate *PM*_10_ forecasts for decision-makers is crucial to protecting public health and preventing potential health risks to the population. Although many studies have attempted to predict particulate matter using various tools (Agarwal & Sahu, 2023; Ahmad & Ahmad, 2023; Folifack Signing et al., 2024; Gul et al., 2022; Khan et al., 2022; Masood & Ahmad, 2023; Rahman & Kabir, 2023; Verma et al., 2023), research specifically predicting future levels of *PM*_10_ in Morocco remains limited. Some studies limit their use to a single station where the models were developed, producing only one forecast for the next hour or the next day (Adnane et al., 2022; Bouakline et al., 2022), or they were only validated at a single station, which limits the generalization of their models to other areas (Ajdour, Leghrib, Chaoufi, Chirmata, et al., 2020; Ajdour, Leghrib, Chaoufi, Fetmaoui, et al., 2020; Saidi et al., 2023).

To develop an effective model for *PM*_10_ prediction, access to ground truth observations is required. Since there are only a few monitoring stations in Morocco with limited temporal coverage (Royaume du Maroc, 2015), using reanalysis data as an alternative form of ground truth provides a viable option (Hassan et al., 2023; Sekmoudi et al., 2021; Vanella et al., 2022), particularly when the ground truth observations are lacking or not available. The reanalysis data, such as those from the Copernicus Atmosphere Monitoring Service (CAMS), create a comprehensive and globally consistent dataset by combining model data with observations around the world, using an atmospheric model based on the laws of physics and chemistry (Inness et al., 2019). While this reanalysis data from CAMS is readily available up to the present, generating future projections remains challenging and poses a significant limitation. On the other hand, we have access to *PM*_10_ forecasts from CAMS global atmospheric composition forecasts for the next 5 days, but these are considered less accurate than the reanalysis data (CAMS, 2021). The challenge here is to bridge the gap between forecasted and reanalyzed data. By correcting these forecasts and bringing them back to the reanalysis, we can provide reliable estimates for future scenarios, even with our inability to directly observe them.

The Analog Ensemble (AnEn) is a model widely applied in forecasting weather events, including wind speed and temperature (Delle Monache et al., 2013), solar power (Alessandrini, Delle Monache, Sperati, & Cervone, 2015), and wind power (Alessandrini, Delle Monache, Sperati, & Nissen, 2015). Its effectiveness has also been demonstrated in improving the forecasts of particulate matter (Delle Monache et al., 2020; Huang et al., 2017; Pappa & Kioutsioukis, 2021; Raman et al., 2021). Therefore, the objective of this study is to implement the AnEn to provide accurate *PM*_10_ forecasts over Morocco for the next 5 days by post-processing CAMS global atmospheric composition forecasts using CAMS reanalysis data. The significance of this research lies in showcasing the effectiveness of AnEn in reducing the gap between the forecasted and reanalyzed data of CAMS, delivering more accurate *PM*_10_ levels with three-hourly forecasts for the upcoming 5 days, contributing to the creation of more reliable forecasts for future events. Additionally, we demonstrate the models’ variations across seasons to make further season-specific adjustments for effective air quality management. This approach also provides *PM*_10_ forecasts that cover all regions in Morocco, which is important for the region that is prone to dust storm events, the main contributors to *PM*_10_ levels (Krasnov et al., 2014). This marks the first application of this approach in Morocco and the broader Middle East and North Africa region.

## Materials and methods

### Source of data

**Fig. 1:**
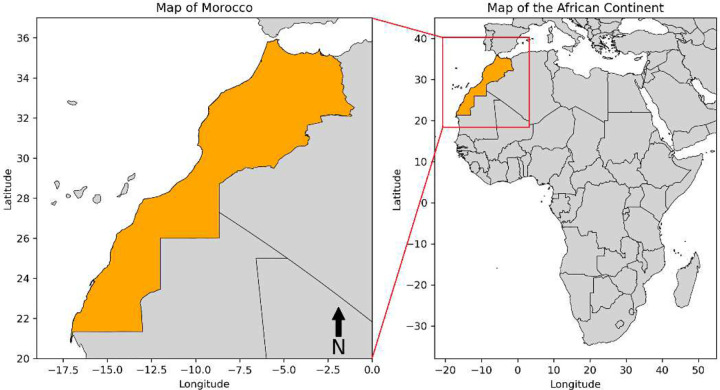
The geographical location of Morocco in North Africa

The *PM*_10_ observations utilized in this study were obtained from CAMS global reanalysis dataset, specifically from EAC4 (ECMWF Atmospheric Composition Reanalysis 4), which is the fourth generation ECMWF global reanalysis of atmospheric composition (Inness et al., 2019). These observations were available at a 3-hourly temporal resolution and a spatial resolution of 0.75°x0.75°. The data, representing the atmospheric conditions over Morocco, were downloaded on 3 February 2023, from the Atmosphere Data Store (ADS) at https://ads.atmosphere.copernicus.eu/cdsapp#!/dataset/cams-global-reanalysis-eac4?tab=overview. *PM*_10_ forecasts, along with forecasts for seven other variables, were derived from CAMS global atmospheric composition forecasts, which generate forecasts up to five days (120 lead time hours) twice a day. The forecasts have a temporal resolution of 1 hour and a spatial resolution of 0.4°x0.4°, covering the entire region of Morocco. Given that pollution in Morocco peaks in the middle of the day, forecasts produced at 12:00 UTC were downloaded in early June 2023 from ADS at https://ads.atmosphere.copernicus.eu/cdsapp\#!/dataset/cams-global-atmosphericcomposition-forecasts?tab=overview. To provide context, the map in Fig. 1 illustrates the geographical location of Morocco in North Africa.

### Data processing

In our study, we transformed the forecasts from 1-hourly data to 3-hourly data by selecting the forecast hours that corresponded to the hours of the observations. As we aim to enhance the accuracy of the forecasts based on the observations, we performed a grid cell regridding operation. We transformed the forecasts from a spatial resolution of 0.4°x0.4° to 0.75°x0.75° by selecting the grid cell centers that matched the observations. The shared temporal range for both the observations and the forecasts was between 21 June 2016, and 30 June 2022. To ensure alignment, we excluded the final five days from the forecasts as they projected beyond the available observation range. This adjustment led to 2196 daily time intervals and 40 lead times in the forecasts, and 17603 3-hourly time intervals in the observations, covering 22 latitude and 23 longitude points, resulting in a total of 506 data points in both datasets. We converted the *PM*_10_ values in both the observations and the forecasts from *kg*/*m*^3^ to *μg*/*m*^3^ for interpretation and visualization purposes. All data processing, preparation, conversion and visualization were carried out using Python 3.8 (Van Rossum & Drake Jr, 1995). The contourf function from the matplotlib.pyplot package (Hunter, 2007) was used for creating maps in Python 3.8.

### Analog ensemble model

The Analog Ensemble (AnEn) technique was developed by (Delle Monache et al., 2013) for weather prediction. AnEn enhances forecasts from a particular model by analyzing historical forecasts produced by that model and past observations. It aims to identify periods when the future target forecast, which represents the current-time forecast at a specific lead time, exhibit values similar to historical forecasts. Once these analogous forecasts are identified, they are considered as analogs to the target forecast. AnEn, having access to both historical forecasts and observations, selects historical observations that align or correspond to these analogs. These selected observations are then averaged into a single value, serving as the corrected new forecast for the initial target forecast. In addition to the target variable, AnEn considers the similarities of forecasts for other variables at the same lead time, comparing them with their historical forecasts, when calculating the closeness of the target forecast to the historical forecasts.

To calculate these similarities, the following metric is used:

$$\left\|F_{t},F_{i}\right\|=\sum_{j=1}^{p}\frac{w_{j}}{\sigma_{j}}\sqrt{\sum_{k=-\tau }^{\tau }{\left(F_{t+k}^{j}-F_{i+k}^{j}\right)}^{2}}$$

Where *F*_*t*_ is the target forecast at lead time *t*, and *F*_*i*_ is the past forecast at a past time *i* in the historical dataset with the same forecast lead time *t*. *p* is the total number of all the variables, including *PM*_10_. *w*_*j*_ is a weight assigned to each variable *j* to provide different weights to different variables. *σ*_*j*_ is the standard deviation of the historical time series of variable *j* at the same forecast lead time *t*. *τ* is half of the number of additional times from which the similarities are calculated. For example, if *τ*=1, then the time window when calculating the metric will be ±3 lead times. $F_{t+k}^{j}$ and $F_{i+k}^{j}$ are the target forecast and the past forecast, respectively, in a time window for a specific variable *j* and lead time *t*.

It is worth noting that AnEn operates independently for each lead time and grid cell. Consequently, the spatial distribution of the data does not influence the model’s decision, unless surrounding historical data are included in the analysis and considered when searching for analogs.

### The correction process using reanalysis data

We applied AnEn to post-process *PM*_10_ forecasts generated from CAMS global forecasts, using the EAC4 global reanalysis dataset as a reference or observations. The data was divided into two parts: a searching set spanning five years from 21 June 2016, to 30 June 2021, and a test set reserved for evaluating the models, covering the period from 01 July 2021, to 25 June 2022. Forecasts of particulate matter (*PM*_2.5_), particulate matter (*PM*_1_), dust aerosol optical depth at 550 nm (AOD), 2m temperature (T), boundary layer height (BLH), U-component of wind (*U*_10_) and V-component of wind (*V*_10_) produced by the CAMS global forecasts model were also included as predictors alongside *PM*_10_.

We conducted a grid search to determine the optimal hyperparameters enhancing the performance of the AnEn model, with a specific focus on optimizing the Root Mean Square Error (RMSE) function. This search involved exploring the number of analogs, with values set at 3, 6, 9, 10, 12, 15, and 21, in addition to assigning weights to each predictor variable, while setting *τ* to 1. Initially, all variables were included with equal weighting (assigned a weight of 1). Then, we proceeded to exclude one variable at a time by setting its weight to 0. Each configuration represented a unique scenario, allowing us to observe how excluding individual variables impacted the model’s performance. This iterative process helped us identify the most influential variables.

Once the optimal model was chosen, we applied bias correction technique (AnEnBc) to enhance AnEn’s forecasting for rare events, assuming linearity between observations and forecasts (Alessandrini et al., 2019). AnEnBc corrects predictions exclusively when the target forecast exceeds a specific threshold. Therefore, various tests were conducted while employing AnEnBc, experimenting with different threshold values: Q25, Q50, MEAN, Q75, Q80, Q85, Q90, and Q95.

AnEnBc adjusts predictions by incorporating a term representing the difference between the target forecast and the average analogs to this forecast. In certain instances, when the target forecast is notably small, its analogs might be significantly larger. Instead of augmenting the predictions, this term will decrease the predictions. When the colinearity between the forecasts and the observations is weak, this correction may potentially end up producing negative values—an undesirable outcome for *PM*_10_. To address this issue, in such cases, we ensure substituting negative values generated by AnEnBc with values from AnEn. This adjustment aims to provide a more coherent output for the model.

The application of AnEn and AnEnBc were carried out using the RAnEn 4.4.6 (Hu et al., 2019) and RAnEnExtra 0.2.13 (Hu et al., 2023) packages in the RStudio software (Posit team, 2023) with R version 4.3.1 (2023-06-16 ucrt) (R Core Team, 2023).

## Results

### Hyperparameter tuning for model optimization

**Fig. 2:**
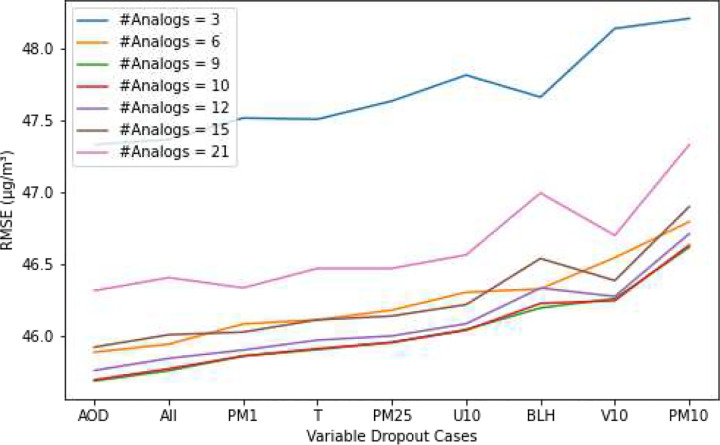
The Results of Grid Search. Root Mean Squared Error (RMSE) Comparison Across Different Configurations. \#Analogs: Number of analogs used in the search. Each case on the X-axis represents the exclusion of one variable from the analysis. *PM*_10_, *PM*_2.5_, and *PM*_1_ stand for particulate matter with diameters less than 10, 2.5, and 1 micrometers, respectively. BLH: Boundary layer height. *U*_10_: U-component of wind. *V*_10_: V-component of wind. T: 2m temperature. AOD: Dust aerosol optical depth at 550 nm. All: Represents the case where all variables are included in the analysis

Fig. 2 shows the grid search results across different tuning hyperparameter configurations. We observe that setting the weight of *PM*_10_ to 0 results in an increase in RMSE across all cases, suggesting that *PM*_10_ is the most influential variable in predictions, followed by *V*_10_, BLH and *U*_10_. This implies that the *PM*_10_ forecasts from CAMS global forecasts play a crucial role in refining themselves. The importance of *V*_10_. BLH and *U*_10_ highlights the significance of atmospheric conditions and wind components in forecasting *PM*_10_ levels. Excluding *PM*_2.5_, 2m temperature, and *PM*_1_ showed a slight increase in RMSE compared to when all variables are included, indicating that these variables have a comparatively lower impact on *PM*_10_ predictions. The reason why atmospheric conditions and wind components have a greater influence on *PM*_10_ is because *PM*_10_ originates predominantly from the Sahara Desert. The winds and boundary layer height (BLH) are the factors that represent the meteorological conditions and weather patterns which contribute to *PM*_10_ levels (Khomsi et al., 2020). While *PM*_2.5_ in Morocco originates mostly from local areas (Khomsi et al., 2020). Given that the major sources of *PM*_10_ and *PM*_2.5_ are different, this may explain why *PM*_2.5_ has a lesser contribution to *PM*_10_ compared to the other meteorological variables. While still relevant, their exclusion doesn’t significantly degrade the model’s performance. Conversely, excluding AOD forecasts leads to a decrease in RMSE, suggesting that it has a limited impact on *PM*_10_ prediction compared to when all variables are included. When examining the impact of the number of analogs used in the search, limiting the search to a very low or high number of analogs results in higher RMSE. This suggests that overly focusing on a small number of analogs may lead to overfitting, while including too many analogs may cause the prediction to deviate from the true forecast value. On the contrary, a moderate value, such as 9 for the number of analogs, yields better generalization in the model.

### Comparative analysis of forecast improvement

Table 1 represents the overall performance of the models. We calculated these performances by reshaping the data from its original dimension, which is four, into one dimension. It is evident from Table 1 that AnEnBc demonstrates superior performance compared to the other models, boasting a MAE of 24.30 *μg*/*m*^3^, RMSE of 44.73 *μg*/*m*^3^, *R*^2^ of 65.18%, Pearson r of 0.822, and a Bias of −9.05 *μg*/*m*^3^. This effectively reduces the overall errors between the predictions and the observations, particularly addressing the largest errors among them as indicated by RMSE. We observe a significant improvement in MAEs in the forecast models, starting from the CAMS forecasts at a MAE of 36.70 *μg*/*m*^3^, improving to 24.44 *μg*/*m*^3^ with AnEn, and further refining to 24.30 *μg*/*m*^3^ with AnEnBc. Likewise, the RMSEs show a similar pattern, beginning at 63.83 *μg*/*m*^3^, then reducing to 45.68 *μg*/*m*^3^ with AnEn, and ultimately reaching 44.73 *μg*/*m*^3^ with AnEnBc. This progression signifies the success of AnEn in correcting the CAMS forecasts and highlights AnEnBc’s role in rectifying the bias present in AnEn for rare events. Additionally, the CAMS forecasts exhibit an *R*^2^ of 29.11%, indicating a limited ability to explain the variability of the observations around their mean. In contrast, AnEnBc demonstrates a more substantial approximation to the observations, achieving an *R*^2^ of 65.18%. The CAMS forecasts reveal a moderate positive correlation of 0.61, indicating a moderate level of the linear relationship between the observations and their forecasts. In contrast, the AnEnBc demonstrates a strong positive correlation, surpassing 0.81, signifying a high degree of the linear relationship between the observations and their predictions.

**Fig. 3:**
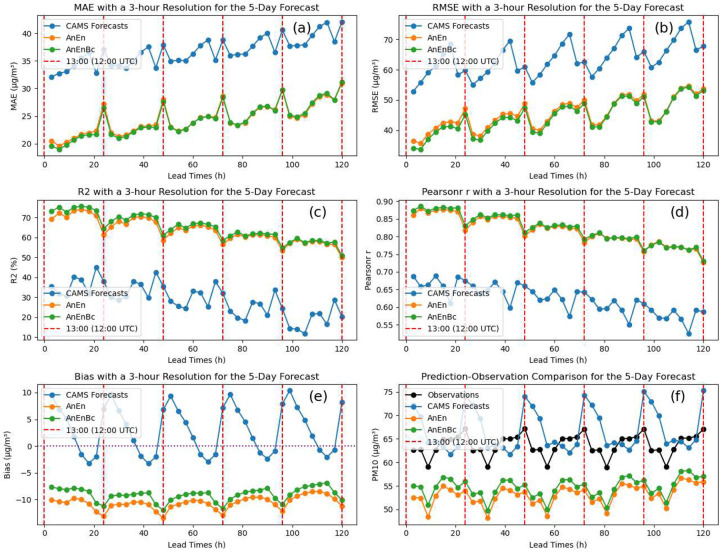
Performance evaluation and prediction-observation comparison with a 3-hour time resolution for the 5-day forecast. CAMS Forecasts: CAMS global atmospheric composition forecasts. AnEn: Analog Ensemble Model. AnEnBc: Bias Correction technique. (a) Mean Absolute Error (MAE) of the models. (b) Root Mean Square Error (RMSE) of the models. (c) Coefficient of determination of the models. (d) Pearson correlation coefficient of the models. (e) Biases of the models. These performances were calculated by reshaping the data from four to two dimensions, while maintaning the dimention of the lead times. (f) A comparison between predictions and observations. This was obtained by averaging the reshaped data at each lead time. The red dashed line is when the CAMS forecasts were produced at 12:00 (UTC)

**Fig. 4:**
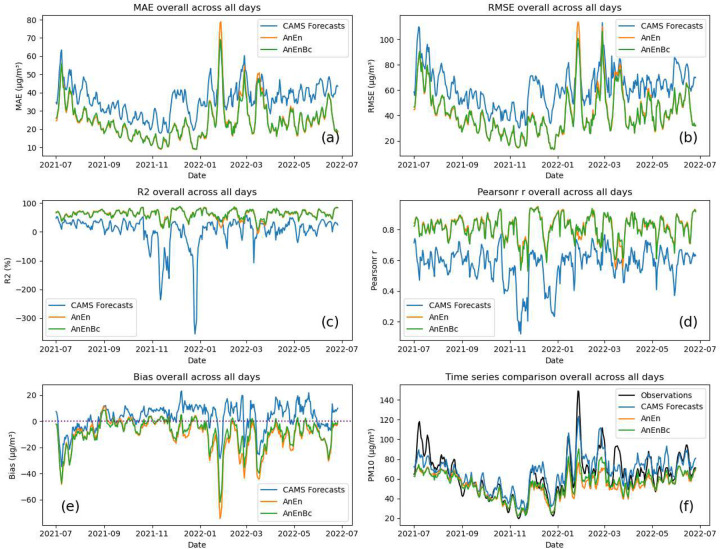
Performance evaluation and prediction-observation comparison overall across all days. CAMS Forecasts: CAMS global atmospheric composition forecasts. AnEn: Analog Ensemble Model. AnEnBc: Bias Correction technique. Observations: CAMS reanalysis data. (a) Mean Absolute Error (MAE) of the models. (b) Root Mean Square Error (RMSE) of the models. (c) Coefficient of determination of the models. (d) Pearson correlation coefficient of the models. (e) Biases of the models. These performances were calculated by reshaping the data from four to two dimensions, while maintaning the dimention of daily times. (f) Time series comparison between predictions and observations. This was obtained by averaging the reshaped data at each day. The evaluation is done on the test set from 01 July 2021, to 25 June 2022

Similarly, when we evaluate the performance of the models at each lead time for the 5-day forecast by examining Fig. 3, we observe significant enhancements in MAE, RMSE, *R*^2^, and Pearson’s r for AnEn and AnEnBc. These improvements are consistent across all lead times, indicating a higher consistency in accurate predictions for both models across all individual observations. In Fig. 4, when evaluating the overall performance of the models across all days, we observe a higher consistency in accurate predictions for AnEn and AnEnBc across all individual observations on each specific day. Except for a few days from 25 January 2023 to 28 January 2023 where the errors of AnEn and AnEnBc are notably higher than those of the CAMS forecasts. This indicates potentially more consistently accurate predictions made by the CAMS forecasts during those days in February. A notable point in Fig. 4 is that the values of *R*^2^ for the CAMS forecasts have reached negative values exceeding −300. *R*^2^ values are supposed to be between 0 and 100 after multiplying by 100. The reason for negative values of *R*^2^ for the CAMS forecasts is because of the failure to reduce the residuals of the model. In some cases, *R*^2^ is defined assuming the total sum of squares of a fitted model is equal to the explained sum of squares plus the residual sum of squares (Miles, 2014). However, the most general definition of *R*^2^ is the difference between 1 and the ratio of the residual sum of squares to the total sum of squares (Di Bucchianico, 2007). When the residual sum of squares is not reduced, negative *R*^2^ can happen. This could be due to a drastic distribution shift from training to testing data or an underperforming model for these specific days in January. When examining the *R*^2^ values for AnEn and AnEnBc, it becomes apparent that the predictions of *PM*_10_ in January have greatly improved, reducing the residuals, and eventually restoring *R*^2^ to its natural values.

When examining the Biases in Table 1, Fig. 3, and Fig. 4, it is clear that the CAMS forecasts exhibit a more favorable Bias of 3.06 *μg*/*m*^3^, better than the other two models. This suggests a similarity in the overall average values between its forecasts and the observations, which is more evident in Fig. 3(f) and Fig. 4(f). In these figures, the overall averages of the CAMS forecasts at each lead time and across all days appear closer to those of the observations, particularly for higher values in Fig. 4. This results in a forecast bias that is more favorable compared to that of the AnEn and AnEnBc models. However, despite this, the lower MAE and RMSE of AnEn and AnEnBc suggest a potentially higher consistency in accurate predictions across all individual observations. To understand the discrepancy between the average closeness and the models’ errors, we conducted a more detailed analysis of the error distribution across all models in Section 3.5. This reveals whether one model consistently has smaller errors or if there are specific patterns causing the difference.

### Spatial evaluation of model predictions

**Fig. 5:**
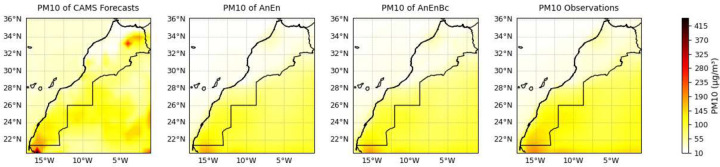
Spatial comparison of predictions and observations. CAMS Forecasts: CAMS global atmospheric composition forecasts. AnEn: Analog Ensemble Model. AnEnBc: Bias Correction technique. Observations: CAMS reanalysis data. These results were obtained by averaging the reshaped data, flattening the lead time and daily time dimensions while maintaining the latitude and longitude dimensions

**Fig. 6:**
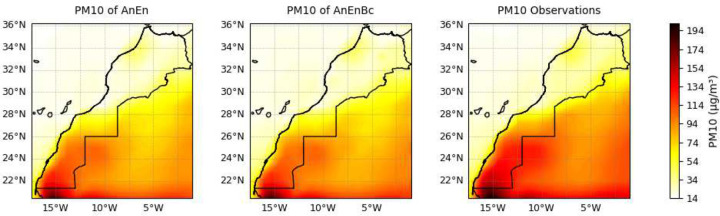
Spatial comparison of predictions and observations, excluding CAMS Forecasts for enhanced visualization. CAMS Forecasts: CAMS global atmospheric composition forecasts. AnEn: Analog Ensemble Model. AnEnBc: Bias Correction technique. Observations: CAMS reanalysis data. These results were obtained by averaging the reshaped data, flattening the lead time and daily time dimensions while maintaining the latitude and longitude dimensions

By calculating the average, as well as the metrics of a reshaped data by flattening the lead times and times dimensions while maintaining the latitude and longitude dimensions, we were able to analyze the spatial distribution of *PM*_10_ as well as the spatial performance of each model. Fig. 5 shows a comparison of the predictions made by each model with the observations. We observe that the AnEn and AnEnBc models significantly contribute to improvements in several areas in Morocco when analyzing the overall average of *PM*_10_ within each grid cell. Starting with the Casablanca-Rabat region in the northwestern, where the CAMS forecasts tend to underestimate the observations, the AnEn and AnEnBc models adeptly capture the patterns occurring in Casablanca-Rabat. Additionally, the CAMS forecasts consistently overestimate many areas, particularly in northeastern Morocco, while the AnEn and AnEnBc models attempt to rectify these inaccuracies. Similar trends are observed in the southern Moroccan desert, where the forecasts show a tendency to significantly overestimate, whereas the AnEn and AnEnBc models perform better in providing more accurate estimations. While the two models try to improve the forecasts, there is a pattern in the southern area indicating higher levels of particulate pollution, something the AnEn and AnEnBc models didn’t capture accurately. Yet, it still highlights the presence of elevated particulate pollution in that region. These higher levels of particulate pollution can be seen more clearly in Fig. 6, where the two models and the observations are normalized separately from the forecasts.

**Fig. 7:**
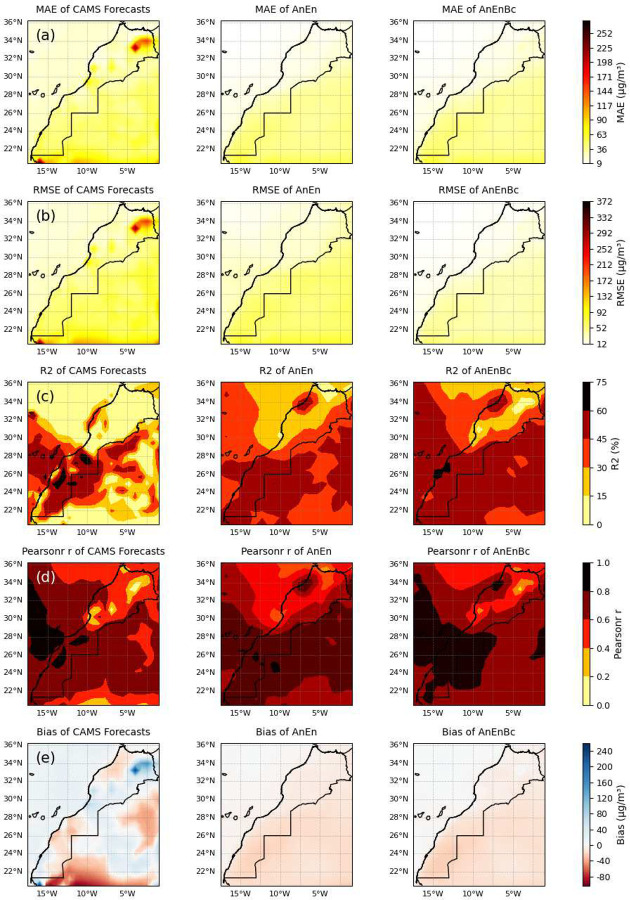
Evaluating the spatial performance of the models overall. CAMS Forecasts: CAMS global atmospheric composition forecasts. AnEn: Analog Ensemble Model. AnEnBc: Bias Correction technique. (a) Mean Absolute Error (MAE) of the models. (b) Root Mean Square Error (RMSE) of the models. (c) Coefficient of determination of the models. (d) Pearson correlation coefficient of the models. (e) Biases of the models. These performances were calculated by reshaping the data, flattening the lead time and daily time dimensions while maintaining the latitude and longitude dimensions

Similarly, when analyzing the spatial performance of each model in Fig. 7, we observe a reduction in MAE and RMSE achieved by AnEn and AnEnBc across all regions in Morocco compared to the CAMS forecasts, demonstrates their consistency in accurately predicting *PM*_10_ levels. The consistency in accurately predicting both overall averages and individual observations within each grid cell emphasizes the spatial robustness of the models. The AnEn and AnEnBc models exhibit significantly high *R*^2^ and correlation with the observations in several areas, notably in the Casablanca-Rabat region, the desert areas in Morocco, and parts of Mauritania and Algeria. This is in contrast to the CAMS forecasts which display lower *R*^2^ values across most areas in Morocco, especially in the North.

The Bias of the CAMS forecasts significantly varies across different areas. In some regions, there is an overestimation of the overall average across all individual observations within each grid cell, while in others, there is a notable underestimation of the overall average of individual observations within each grid cell. In contrast, the AnEn and AnEnBc significantly reduce these Biases to moderate levels around 0, implying that the two AnEn models provide more balanced and unbiased predictions, aligning more closely with observations across different regions.

### Evaluating the models’ performance in each season

Now, instead of evaluating the performance of each model overall across all seasons, we assessed their performance in each season. Table 2 reveals that the overall progress made by AnEn seems consistent across each season. Applying bias correction (AnEnBc) further improves errors in spring and winter. However, when applying AnEnBc in summer and autumn, it appears to worsen predictions rather than improve them. This is likely due to the higher presence of non-linearity between the observations and the CAMS forecasts in these two seasons compared to spring and winter. Since AnEnBc only improves the predictions of rare events when the linear relations assumption is fulfilled (Alessandrini et al., 2019).

A similar finding was noted in Online Resource Figures S1, S2, S3 and S4, where the overall progress made by AnEn and AnEnBc at each lead time seems consistent across each season. When analyzing the performance at each lead time in each season, it becomes more obvious that AnEnBc in spring and winter shows a significant improvement, especially in winter for specific lead times. However, in summer and autumn, this improvement diminishes, and the performance of AnEnBc becomes similar to that of AnEn, and in some lead times, it even appears worse, as identified in Table 2.

Observing the *PM*_10_ values across different seasons in Online Resource Fig. S5 reveals a consistent pattern similar to the overall trend in Fig. 5. However, the presence of elevated particulate pollution becomes notably more pronounced in the summer and winter seasons. This could indicate a limitation in the AnEn’s ability to capture the overall averages in these desert regions with specific environmental conditions. However, upon examining the errors of the forecasts in Online Resource Figures S6 and S7, it becomes evident that AnEn and AnEnBc notably enhance both the MAE and RMSE, particularly in the desert area and northeastern Morocco across all seasons. Consistent with earlier findings, despite the CAMS forecasts achieving high *R*^2^ values or strong correlations, AnEn and AnEnBc consistently outperform them across most regions of Morocco in all seasons. Similarly, the bias of the CAMS forecasts is inconsistent even within each season. As AnEn and AnEnBc enhance the bias of the forecasts, it becomes more apparent that they consistently underestimate *PM*_10_ on average in the desert area.

### Explaining the discrepancy between errors and bias

**Fig. 8:**
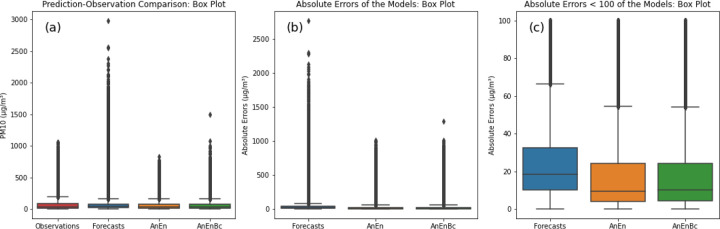
Comparisons of predictions-observations and absolute errors across all models (Box Plots). Forecasts: CAMS global atmospheric composition forecasts. AnEn: Analog Ensemble Model. AnEnBc: Bias Correction technique. Observations: CAMS reanalysis data. (a) Box plot showing predictions and observations. (b) Box plot illustrating absolute differences between predictions and observations. (c) Box plot illustrating absolute differences less than 100 μg/m3 between predictions and observations

To determine whether a particular model consistently has smaller errors or if there are specific patterns causing the difference and discrepancy between the average closeness and the models’ errors, we conducted further analysis of the distribution of *PM*_10_, along with examining the error distribution generated by each model. In Fig. 8(a), which shows the distribution of *PM*_10_ for each model using a Box plot, we observe extremely heavy outliers produced by the CAMS forecasts, surpassing both the outliers of the observations and those of AnEn and AnEnBc. They even exceed the observations’ outliers by threefold. The presence of these outliers and extreme values could heavily influence and deviate the overall average of the CAMS forecasts, potentially bringing it closer to the observations.

**Fig. 9:**
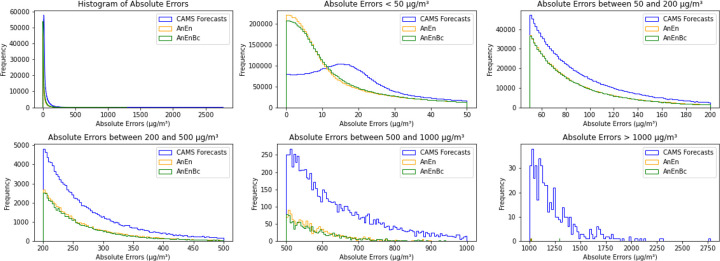
Histogram of absolute errors between predictions and observations. CAMS Forecasts: CAMS global atmospheric composition forecasts. AnEn: Analog Ensemble Model. AnEnBc: Bias Correction technique

**Fig. 10:**
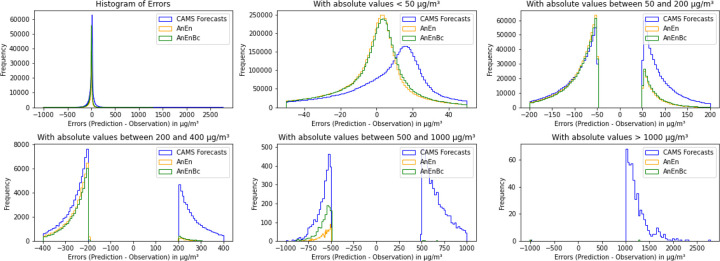
Histogram of errors: differences between predictions and observations. CAMS Forecasts: CAMS global atmospheric composition forecasts. AnEn: Analog Ensemble Model. AnEnBc: Bias Correction technique

When looking at Fig. 8(b), where the box plot of the absolute errors is further plotted, it is evident that the CAMS forecasts exhibit errors exceeding 1000 *μg*/*m*^3^, surpassing those of AnEn and AnEnBc. To see the exact distribution of these errors, we plotted only the absolute errors with values less than 100 *μg*/*m*^3^ in Fig. 8(c), revealing that 25% of the CAMS forecasts’ absolute errors are less than 10 *μg*/*m*^3^, while 50% of AnEn and AnEnBc models’ absolute errors fall below 10 *μg*/*m*^3^. This explains the substantial lack of accuracy in the CAMS forecasts. Additionally, fewer than 50% of the CAMS forecasts’ absolute errors are less than 20 *μg*/*m*^3^, contrasting with over 70% of AnEn and AnEnBc models’ absolute errors being below 20 *μg*/*m*^3^. This consistent trend demonstrates that the two AnEn and AnEnBc models consistently generate smaller errors across all predictions, resulting in a lower MAE and RMSE compared to the CAMS forecasts. Fig. 9 demonstrates further the exact distributions of the absolute errors across all the models. In this figure, it is evident that most of the errors of the AnEn and AnEnBc are close to 0, while most of the errors of the CAMS forecasts are far from 0, especially the absolute errors exceeding 500 *μg*/*m*^3^ and reaching 2700 *μg*/*m*^3^. Likewise, in Fig. 10, the majority of errors (predictions - observations) are centered around 0. However, as we move away from 0, the frequency distribution of errors in the CAMS forecasts is notably higher than those of the AnEn and AnEnBc, particularly the positive errors, indicating a systematic issue in the CAMS global forecasting model. This reveals that even though the CAMS forecasts might exhibit a lower bias due to their closer alignment with observed values on average, only a few predictions closely resemble observations while many others significantly deviate.

In addition to what has been said, the CAMS forecasts exhibit inconsistency in overestimating and underestimating across different areas and times, resulting in both extremely high and low biases, especially as shown in Fig. 7(e). Averaging these biases, the final bias, which represents the overall bias across all individual observations, could be considerably smaller than that of AnEn and AnEnBc. This explains further why the CAMS forecasts align more closely with the observations when considering the overall averages. Furthermore, the dataset’s variability might also significantly contribute to the divergence between the AnEn and AnEnBc averages and that of the observations, particularly because both the CAMS forecasts and the observations demonstrate greater variability in comparison to the AnEn and AnEnBc, as shown in Online Resource Table S1.

## Discussion

In this study, we refined *PM*_10_ forecasts produced from CAMS global atmospheric composition forecasts over Morocco for the next 5 days using CAMS reanalysis data with the Analog Ensemble Model (AnEn). Bias correction (AnEnBc) was applied to correct biases in AnEn for rare events. This marks the first application of such an approach in Morocco and the Middle East and North Africa region. The results show that both models demonstrate superior performance compared to the CAMS forecasts. Enhancements in MAE, RMSE, R2, and Pearson’s R for the AnEn and AnEnBc models suggest a higher consistency in accurate predictions across all temporal and spatial observations.

The enhancement achieved by AnEn results from its success in capturing relevant observations from the past that align with the current forecast model (Delle Monache et al., 2013). Both AnEn and AnEnBc models significantly contributed to improvements across various areas in Morocco, demonstrating their effectiveness in forecasting *PM*_10_ in different land use, topographical complexities, and geographic regions. This effectiveness was also demonstrated by (Delle Monache et al., 2020), where spatial distribution improvement in RMSE for particulate matter and ozone was highlighted. Consistent results were also observed (Golbazi et al., 2024; Solomou et al., 2021), emphasizing the models’ effectiveness in enhancing forecasts for particulate matter, including *PM*_10_ and *PM*_2.5_. The observed increase in MAE and RMSE errors in Fig. 3, as the lead time extends, is likely attributed to the uncertainties inherent in a longer forecasting period. This phenomenon is common in various forecasting events, such as atmospheric rivers’ water vapor signature forecasts (Wick et al., 2013), making it challenging to accurately predict future demand.

The enhancements in MAE and RMSE of *PM*_10_ achieved by AnEn across different seasons reveal a consistent pattern similar to the improvement made in the overall trend. Similar outcomes are demonstrated in (Solomou et al., 2021), where AnEn techniques successfully reduce RMSE values for all seasons, addressing both particulate matter pollutants (*PM*_10_ and *PM*_2.5_). Likewise, Pappa and Kioutsioukis (2021) illustrates that AnEn consistently improves the RMSE of CAMS forecasts across all seasons, suggesting that the progress made by AnEn is not confined to a specific season but extends across different seasons. AnEnBc exhibits a significant improvement over AnEn, specifically in the spring and winter seasons where high values of *PM*_10_ are located (Fig. 4(f)), in contrast to other seasons like summer and autumn where *PM*_10_ values are comparatively lower, specifically in autumn. This improvement of AnEnBc is more evident when analyzing the performance at each lead time in Online Resource Figures S1(d), S2(d), S3(d) and S4(d), and the overall performance of the models in Table 2. AnEnBc generally improves predictions, especially in cases where the forecasts are higher (Alessandrini et al., 2019). While this improvement is not pronounced for high values of *PM*_10_ in the summer, it is distinctly visible for extreme values of *PM*_10_ in both spring and winter seasons. These variations in AnEnBc’s behavior across different seasons may be influenced by several factors, including meteorological conditions and the source emissions of *PM*_10_. These fluctuations could also be attributed to the higher presence of non-linearity between observations and CAMS forecasts, potentially stemming from modeling defects and the absence of accurate emission inventories in CAMS forecasts (H. Liu et al., 2021; Varga-Balogh et al., 2020; T. Zhang et al., 2020). This may also explain the errors in CAMS forecasts in the northeastern and desert areas in the south. Additionally, the accuracy of particulate matter forecasts also hinges on optimizing the physical and chemical theories underlying the forecasting model (Bai et al., 2018; Feng et al., 2015), which can pose challenges and be difficult to perform (Wu et al., 2020). Furthermore, the exceptional overestimation of *PM*_10_ from CAMS forecasts could also be linked to events of Sahara dust transport (Pappa & Kioutsioukis, 2021), which occur often in the Saharan areas, especially in southern Morocco.

Despite the enhancements made by AnEn and AnEnBc, accurately predicting high extreme values remains challenging, particularly evident in the elevated particulate pollution in the southern Moroccan desert, occurring more notably in the summer (Online Resource Fig. S5), and also in February and March when high *PM*_10_ events occur, as seen in Figures 4(a), 4(b), and 4(c). While Delle Monache et al. (2013) and Alessandrini et al. (2019) indicate that the AnEn model performs relatively well for extreme events, showcasing lower RMSE and higher correlation coefficients under specific conditions for 10-m wind speed, particulate matter, and ozone, challenges and limitations of the AnEn model are also emphasized in (Delle Monache et al., 2020) and (Huang et al., 2017), especially in handling extremely high concentration events of *PM*_2.5_. The variability in AnEn performance can be attributed to several factors, as noted in previous studies. Alessandrini et al. (2019) highlight a reduction in available quality analogs during extreme events, suggesting that longer training periods may enhance AnEn’s performance. On the other hand, Huang et al. (2017) argues that certain events, such as Independence Day fireworks, wildfires and wind-blown dust episodes, present challenges for accurate predictions due to the nature of the analog selection process. These insights underscore the complexity of capturing diverse environmental conditions with analog methods. Additionally, it’s worth noting that the effectiveness of the analog ensemble method may vary depending on the stability of climate or air pollution patterns (Hamill & Whitaker, 2006). While challenges exist, recognizing and addressing these limitations can lead to further refinements in utilizing analogs for improved forecasting accuracy.

Refining CAMS atmospheric composition forecasts using reanalysis data is considered a significant step towards achieving improved accuracy and reliability in predicting particulate matter levels in Morocco. The corrections introduced by the analog ensemble model, learning from past historical observations in reanalysis, demonstrate its superior performance in terms of MAE, RMSE, *R*^2^, and Pearson’s r, positioning itself as a valuable tool for improving the precision of CAMS forecasts. This refinement not only diminishes errors in *PM*_10_ predictions but also ensures spatial consistency, thereby highlighting its potential translation into early and more accurate warnings of high *PM*_10_ pollution events. Applying such methodologies to environmental policies and public health decision-making can minimize the health impacts associated with air pollution. Recognizing the models’ variations across seasons further allows for season-specific adjustments in forecasting strategies, contributing to more effective air quality management. Moreover, accurate forecasting of *PM*_10_ helps mitigate the risks and impacts of dust storm events. This is because of the easy movement and transportation of dust particles contained in *PM*_10_ during these dust storms (Q. Liu et al., 2014). These dust storms not only pose a threat to air quality but also to public health, environmental sustainability, and various economic sectors (Middleton & Kang, 2017). Therefore, by refining *PM*_10_ forecasts, especially in regions where dust storms are dominant or occur often in Morocco, authorities and stakeholders can better prepare for and respond to these events.

While the paper significantly improves CAMS *PM*_10_ forecasts, aligning them more closely with reanalysis data, it is essential to acknowledge a limitation in the generalizability of findings to ground truth observations. This limitation arises from the reliance on ground-level data from CAMS reanalysis as a reference, a necessary approach given the absence of data from monitoring stations. Despite this constraint, the study provides valuable insights into the improved forecast accuracy facilitated by AnEn, offering a robust framework for enhancing *PM*_10_ predictions in atmospheric composition forecasts. As a suggestion for future research, considering other bias correction tools and models could be explored for enhancing rare high events of particulate matter in Morocco. Testing alternative techniques like machine learning and deep learning models may also be considered useful tools for further improving *PM*_10_ forecasts. Additionally, more studies in Morocco are needed to reveal the most crucial variables contributing to elevated pollution in Morocco and the scenarios leading to these situations. This information is crucial for engineers and data scientists to focus more on these important predictors and further improve the prediction of high pollution values.

## Conclusions

In this study, we explored the use of the analog ensemble (AnEn) model and the bias correction (AnEnBC) technique to provide accurate *PM*_10_ forecasts over Morocco by post-processing CAMS *PM*_10_ forecasts, using CAMS reanalysis data as a reference. This is the first application of such an approach in Morocco and the broader Middle East and North Africa. The results show substantial prediction improvements in MAE, RMSE, *R*^2^, and Pearson’s r, reducing the MAE and RMSE of the CAMS forecasts by 12 *μg*/*m*^3^ and 19 *μg*/*m*^3^, respectively. This enhancement suggests a higher consistency in accurate predictions across all temporal and spatial observations of *PM*_10_. Refining CAMS atmospheric composition forecasts is considered a significant step towards achieving improved accuracy and reliability in predicting particulate matter levels in Morocco. The application of these approaches to environmental policies and public health decision-making can contribute to more effective air quality management and eventually minimize air pollution health impacts.

## Figures and Tables

**Table 1:** Evaluating the Performance of the Models Overall

	MAE (*μg/m*^3^)	RMSE (*μg/m*^3^)	*R*^*2*^ (%)	Parsonr r	Bias (*μg/m*^*3*^)
AnEnBc	24,30974*	44,73886*	65,18205*	0,822434*	−9,05514
AnEn	24,44007	45,68253	63,69774	0,817895	−10,619
CAMS Forecasts	36,70845	63,83657	29,11204	0,619938	3,069687*

MAE: Mean Absolute Error. RMSE: Root Mean Square Error. *R*^2^: Coefficient of Determination. Pearson r: Pearson Correlation Coefficient. Bias: The difference between predictions and observations. AnEn: Analog Ensemble Model. AnEnBc: Bias Correction technique. CAMS Forecasts: CAMS global atmospheric composition forecasts.

* indicates the best-performing model.

**Table 2:** Evaluating the Models’ Overall Performance in Each Season

Seasons	Models	MAE (*μg/m*^3^)	RMSE (*μg/m*^3^)	*R*^*2*^ (%)	Parsonr r	Bias (*μg/m*^3^)
Spring	AnEnBc	25,86968*	46,22565*	61,06998*	0,795237*	−9,89951
	AnEn	26,16834	47,53721	58,82953	0,789684	−12,4868
	CAMS Forecasts	39,10532	66,70214	18,94161	0,597938	4,540903*
Summer	AnEnBc	29,54205	53,54986	63,74854	0,820088	−13,4031
	AnEn	28,98313*	52,99216*	64,49969*	0,82413*	−12,9379
	CAMS Forecasts	41,56744	72,71678	33,15359	0,612462	−4,34543*
Autumn	AnEnBc	16,76254	30,13316	68,64954	0,830115	−1,80822
	AnEn	16,5572*	29,91776*	69,09614*	0,832457*	−1,66406
	CAMS Forecasts	27,37132	48,34186	19,3134	0,591566	5,385614*
Winter	AnEnBc	25,28831*	46,17216*	65,36883*	0,832884*	−11,3164
	AnEn	26,25224	49,17304	60,72095	0,820021	−15,5226
	CAMS Forecasts	39,00215	65,40652	30,50576	0,640465	6,392065*

MAE: Mean Absolute Error. RMSE: Root Mean Square Error. *R*^2^: Coefficient of Determination. Pearson r: Pearson Correlation Coefficient. Bias: The difference between predictions and observations. AnEn: Analog Ensemble Model. AnEnBc: Bias Correction technique. CAMS Forecasts: CAMS global atmospheric composition forecasts.

* indicates the best-performing model.

## Data Availability

The CAMS global reanalysis data (Inness et al., 2019) and CAMS global atmospheric composition forecasts used in the study are available at the Copernicus Atmosphere Monitoring Service (CAMS) and were downloaded from the Atmosphere Data Store (ADS) (https://ads.atmosphere.copernicus.eu/cdsapp#!/dataset/cams-global-reanalysis-eac4?tab=overview and https://ads.atmosphere.copernicus.eu/cdsapp#!/dataset/cams-global-atmospheric-composition-forecasts?tab=overview). Access to the data requires registration, signing in, and submitting a request, in compliance with the Copernicus data license (https://cds.climate.copernicus.eu/api/v2/terms/static/licence-to-use-copernicus-products.pdf).
